# Supplementary material for: Transcriptome Analysis Reveals Vimentin-Induced Disruption of Cell–Cell Associations Augments Breast Cancer Cell Migration
Source: Cells. 2022 Dec 13;11(24):4035. doi: 10.3390/cells11244035 (PMC9776984; doi:10.3390/cells11244035)
Supplement: Supplementary file 1 [file cells-11-04035-s001.zip › DEGs and enrichment analysis.zip!/DEGs and enrichment analysis/WT_VIMvsVector GO enrichment/GSEA/WT_VIM/LENS_DEVELOPMENT_IN_CAMERA_TYPE_EYE/LENS_DEVELOPMENT_IN_CAMERA_TYPE_EYE(GO_0002088).html]

Details for gene set LENS\_DEVELOPMENT\_IN\_CAMERA\_TYPE\_EYE(GO:0002088)[GSEA]


|  || Dataset | fpkm.sample |
| Phenotype | sample.cls |
| Upregulated in class | WT\_VIM |
| GeneSet | LENS\_DEVELOPMENT\_IN\_CAMERA\_TYPE\_EYE(GO:0002088) |
| Enrichment Score (ES) | 0.92374974 |
| Normalized Enrichment Score (NES) | 1.4728954 |
| Nominal p-value | 0.0 |
| FDR q-value | 0.1629998 |
| FWER p-Value | 0.163 |
Table: GSEA Results Summary

  

Fig 1: Enrichment plot: LENS\_DEVELOPMENT\_IN\_CAMERA\_TYPE\_EYE(GO:0002088)
  
Profile of the Running ES Score & Positions of GeneSet Members on the Rank Ordered List

  

| PROBE | DESCRIPTION   (from dataset) | GENE SYMBOL | GENE\_TITLE | RANK IN GENE LIST | RANK METRIC SCORE | RUNNING ES | CORE ENRICHMENT || 1 | ENSG00000107859 | PITX3 |  |  | 641 | Infinity | -0.0108 | Yes |
| 2 | ENSG00000138083 | SIX3 |  |  | 1590 | Infinity | -0.0269 | Yes |
| 3 | ENSG00000105370 | LIM2 |  |  | 1834 | Infinity | -0.0310 | Yes |
| 4 | ENSG00000105989 | WNT2 |  |  | 1850 | Infinity | -0.0312 | Yes |
| 5 | ENSG00000026025 | VIM |  |  | 2388 | 1709.539 | 0.9237 | Yes |
| 6 | ENSG00000186790 | FOXE3 |  |  | 3327 | 3.955 | 0.9101 | No |
| 7 | ENSG00000140538 | NTRK3 |  |  | 3371 | 3.836 | 0.9115 | No |
| 8 | ENSG00000111186 | WNT5B |  |  | 3829 | 3.244 | 0.9056 | No |
| 9 | ENSG00000135517 | MIP |  |  | 4670 | 2.577 | 0.8929 | No |
| 10 | ENSG00000135446 | CDK4 |  |  | 4816 | 2.481 | 0.8918 | No |
| 11 | ENSG00000110400 | NECTIN1 |  |  | 4962 | 2.427 | 0.8907 | No |
| 12 | ENSG00000170819 | BFSP2 |  |  | 6181 | 1.992 | 0.8712 | No |
| 13 | ENSG00000125378 | BMP4 |  |  | 6326 | 1.969 | 0.8699 | No |
| 14 | ENSG00000176887 | SOX11 |  |  | 6473 | 1.930 | 0.8685 | No |
| 15 | ENSG00000109846 | CRYAB |  |  | 7118 | 1.789 | 0.8586 | No |
| 16 | ENSG00000105329 | TGFB1 |  |  | 8097 | 1.609 | 0.8430 | No |
| 17 | ENSG00000105325 | FZR1 |  |  | 8243 | 1.586 | 0.8414 | No |
| 18 | ENSG00000134245 | WNT2B |  |  | 8875 | 1.496 | 0.8316 | No |
| 19 | ENSG00000108255 | CRYBA1 |  |  | 8890 | 1.494 | 0.8322 | No |
| 20 | ENSG00000107485 | GATA3 |  |  | 9538 | 1.412 | 0.8220 | No |
| 21 | ENSG00000111276 | CDKN1B |  |  | 9723 | 1.392 | 0.8197 | No |
| 22 | ENSG00000188064 | WNT7B |  |  | 9943 | 1.366 | 0.8167 | No |
| 23 | ENSG00000168036 | CTNNB1 |  |  | 12639 | 1.129 | 0.7718 | No |
| 24 | ENSG00000184564 | SLITRK6 |  |  | 12695 | 1.125 | 0.7715 | No |
| 25 | ENSG00000137936 | BCAR3 |  |  | 12717 | 1.123 | 0.7717 | No |
| 26 | ENSG00000186575 | NF2 |  |  | 12952 | 1.104 | 0.7684 | No |
| 27 | ENSG00000064309 | CDON |  |  | 13001 | 1.102 | 0.7682 | No |
| 28 | ENSG00000163348 | PYGO2 |  |  | 13473 | 1.066 | 0.7608 | No |
| 29 | ENSG00000157933 | SKI |  |  | 13763 | 1.045 | 0.7565 | No |
| 30 | ENSG00000125686 | MED1 |  |  | 13965 | 1.029 | 0.7537 | No |
| 31 | ENSG00000146950 | SHROOM2 |  |  | 15281 | 0.953 | 0.7320 | No |
| 32 | ENSG00000177045 | SIX5 |  |  | 16096 | 0.913 | 0.7187 | No |
| 33 | ENSG00000136842 | TMOD1 |  |  | 16244 | 0.903 | 0.7167 | No |
| 34 | ENSG00000143995 | MEIS1 |  |  | 16354 | 0.896 | 0.7154 | No |
| 35 | ENSG00000163349 | HIPK1 |  |  | 16484 | 0.889 | 0.7137 | No |
| 36 | ENSG00000142627 | EPHA2 |  |  | 16692 | 0.877 | 0.7107 | No |
| 37 | ENSG00000125875 | TBC1D20 |  |  | 17195 | 0.845 | 0.7027 | No |
| 38 | ENSG00000164442 | CITED2 |  |  | 17224 | 0.843 | 0.7027 | No |
| 39 | ENSG00000166225 | FRS2 |  |  | 17696 | 0.816 | 0.6952 | No |
| 40 | ENSG00000007372 | PAX6 |  |  | 18507 | 0.771 | 0.6819 | No |
| 41 | ENSG00000040531 | CTNS |  |  | 19688 | 0.706 | 0.6623 | No |
| 42 | ENSG00000114251 | WNT5A |  |  | 19962 | 0.691 | 0.6581 | No |
| 43 | ENSG00000146350 | TBC1D32 |  |  | 20667 | 0.652 | 0.6465 | No |
| 44 | ENSG00000125864 | BFSP1 |  |  | 21341 | 0.617 | 0.6355 | No |
| 45 | ENSG00000075711 | DLG1 |  |  | 21584 | 0.603 | 0.6317 | No |
| 46 | ENSG00000151012 | SLC7A11 |  |  | 21597 | 0.602 | 0.6319 | No |
| 47 | ENSG00000064393 | HIPK2 |  |  | 21817 | 0.587 | 0.6285 | No |
| 48 | ENSG00000117139 | KDM5B |  |  | 22622 | 0.535 | 0.6152 | No |
| 49 | ENSG00000106799 | TGFBR1 |  |  | 22648 | 0.534 | 0.6150 | No |
| 50 | ENSG00000163499 | CRYBA2 |  |  | 23113 | 0.498 | 0.6075 | No |
| 51 | ENSG00000177707 | NECTIN3 |  |  | 23225 | 0.494 | 0.6059 | No |
| 52 | ENSG00000196116 | TDRD7 |  |  | 23479 | 0.480 | 0.6019 | No |
| 53 | ENSG00000136603 | SKIL |  |  | 23480 | 0.480 | 0.6021 | No |
| 54 | ENSG00000178573 | MAF |  |  | 24185 | 0.422 | 0.5904 | No |
| 55 | ENSG00000166949 | SMAD3 |  |  | 24688 | 0.383 | 0.5822 | No |
| 56 | ENSG00000163513 | TGFBR2 |  |  | 24808 | 0.372 | 0.5804 | No |
| 57 | ENSG00000188158 | NHS |  |  | 25709 | 0.281 | 0.5653 | No |
| 58 | ENSG00000152661 | GJA1 |  |  | 25762 | 0.276 | 0.5646 | No |
| 59 | ENSG00000213139 | CRYGS |  |  | 26210 | 0.228 | 0.5571 | No |
| 60 | ENSG00000119608 | PROX2 |  |  | 26920 | 0.127 | 0.5452 | No |
| 61 | ENSG00000169554 | ZEB2 |  |  | 27993 | 0.000 | 0.5270 | No |
| 62 | ENSG00000154764 | WNT7A |  |  | 31003 | NaN | 0.4761 | No |
| 63 | ENSG00000141161 | UNC45B |  |  | 37244 | NaN | 0.3705 | No |
| 64 | ENSG00000118231 | CRYGD |  |  | 46052 | NaN | 0.2214 | No |
| 65 | ENSG00000203733 | GJE1 |  |  | 50942 | NaN | 0.1386 | No |
| 66 | ENSG00000182187 | CRYGB |  |  | 51074 | NaN | 0.1364 | No |
| 67 | ENSG00000121634 | GJA8 |  |  | 51825 | NaN | 0.1237 | No |
| 68 | ENSG00000117707 | PROX1 |  |  | 53003 | NaN | 0.1038 | No |
| 69 | ENSG00000182968 | SOX1 |  |  | 58105 | NaN | 0.0174 | No |
| 70 | ENSG00000168582 | CRYGA |  |  | 58439 | NaN | 0.0118 | No |
Table: GSEA details
[plain text format]

  

Fig 2: LENS\_DEVELOPMENT\_IN\_CAMERA\_TYPE\_EYE(GO:0002088)
  
Blue-Pink O' Gram in the Space of the Analyzed GeneSet

  

Fig 3: LENS\_DEVELOPMENT\_IN\_CAMERA\_TYPE\_EYE(GO:0002088): Random ES distribution
  
Gene set null distribution of ES for
 **LENS\_DEVELOPMENT\_IN\_CAMERA\_TYPE\_EYE(GO:0002088)**
